# Supplementary material for: Establishment of Repeated In Vitro Exposure System for Evaluating Pulmonary Toxicity of Representative Criteria Air Pollutants Using Advanced Bronchial Mucosa Models
Source: Toxics. 2022 May 24;10(6):277. doi: 10.3390/toxics10060277 (PMC9228979; doi:10.3390/toxics10060277)
Supplement: Supplementary file 1 [file toxics-10-00277-s001.zip › toxics-1691391-supplementary.pdf]

toxics

MDPI

# Supplementary Materials: Establishment of Repeated In Vitro Exposure System for Evaluating Pulmonary Toxicity of Representative Criteria Air Pollutants Using Advanced Bronchial Mucosa Models

**Swapna Upadhyay, Ashesh Chakraborty, Tania A. Thimraj, Marialuisa Baldi, Anna Steneholm, Koustav Ganguly, Per Gerde, Lena Ernstgård and Lena Palmberg**

**1. Repeated gas ($NO_2$ and $SO_2$) exposure induced pro-inflammatory, oxidative stress and tissue injury/repair effect on bro-ALI:**

*1. a. Transcript expression of pro-inflammatory, oxidative stress, and tissue injury/repair markers:*

Figure S1, S2 and S3 depicts transcript expression of pro-inflammatory, oxidative stress and tissue injury/repair markers following repeated exposure to low and high concentration of gaseous ($NO_2$ and $SO_2$) air pollutants. Most of these markers is showing reduced transcript expression at 48 h and 72 h after exposure to both low and high gas exposure compared to sham and 24 h post exposure, however none of these markers were statistically significant. except transcript expression of *MMP9* at 48 and 72 h (Figure S3) high gas exposure compared to bro-ALI at the 24 h exposure to high gaseous air pollutant.

*1. b. Protein concentration of pro-inflammatory, and tissue injury/repair markers:*

Concentration of IL8 and MMP9 protein in BM were significantly reduced mostly in all time points (24, 48 and 72 h) after both low and high gas exposure, except significantly increased concentration of IL8 was measured at 24 after high gas exposure (Figure S4).

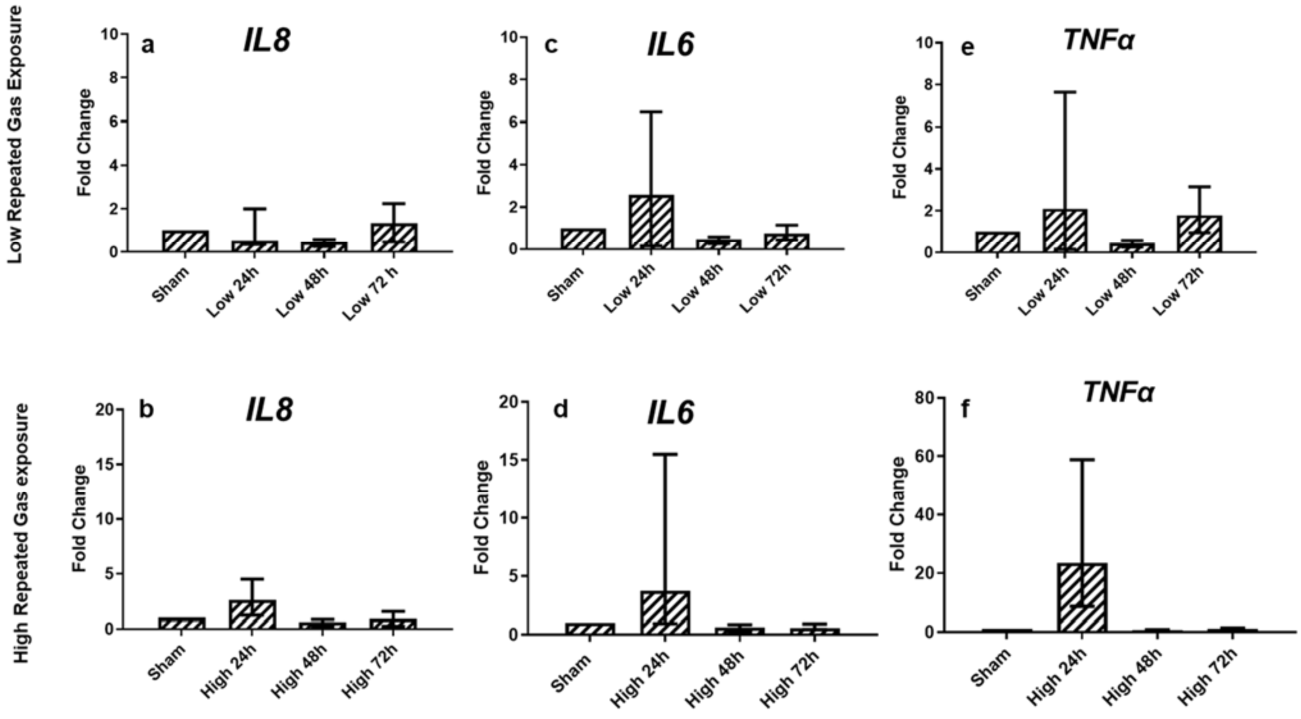


**Figure S1. mRNA expression of inflammatory markers following repeated exposure to low and high doses of nitrogen dioxides ($NO_2$) and sulphur dioxides ($SO_2$).** Fold change *of IL8, IL6, and TNFα* in bronchial mucosa models developed at air-liquid interface (bro-ALI) following incubation of 24 hours (h) after 30 minutes exposure to sham (control: clean air exposure) or gases :low ($NO_2$: 0.1 ppm; $SO_2$: 0.2 ppm) and high ($NO_2$: 0.2 ppm; $SO_2$: 0.4 ppm) for 3 time points (24, 48 and 72 h). Data presented as median and 25$^{th}$ -75$^{th}$ percentiles, fold change =2-ΔCt of models / 2-ΔCt of sham exposed bro-ALI. N=4 donors and n=2 replicates/Donor.

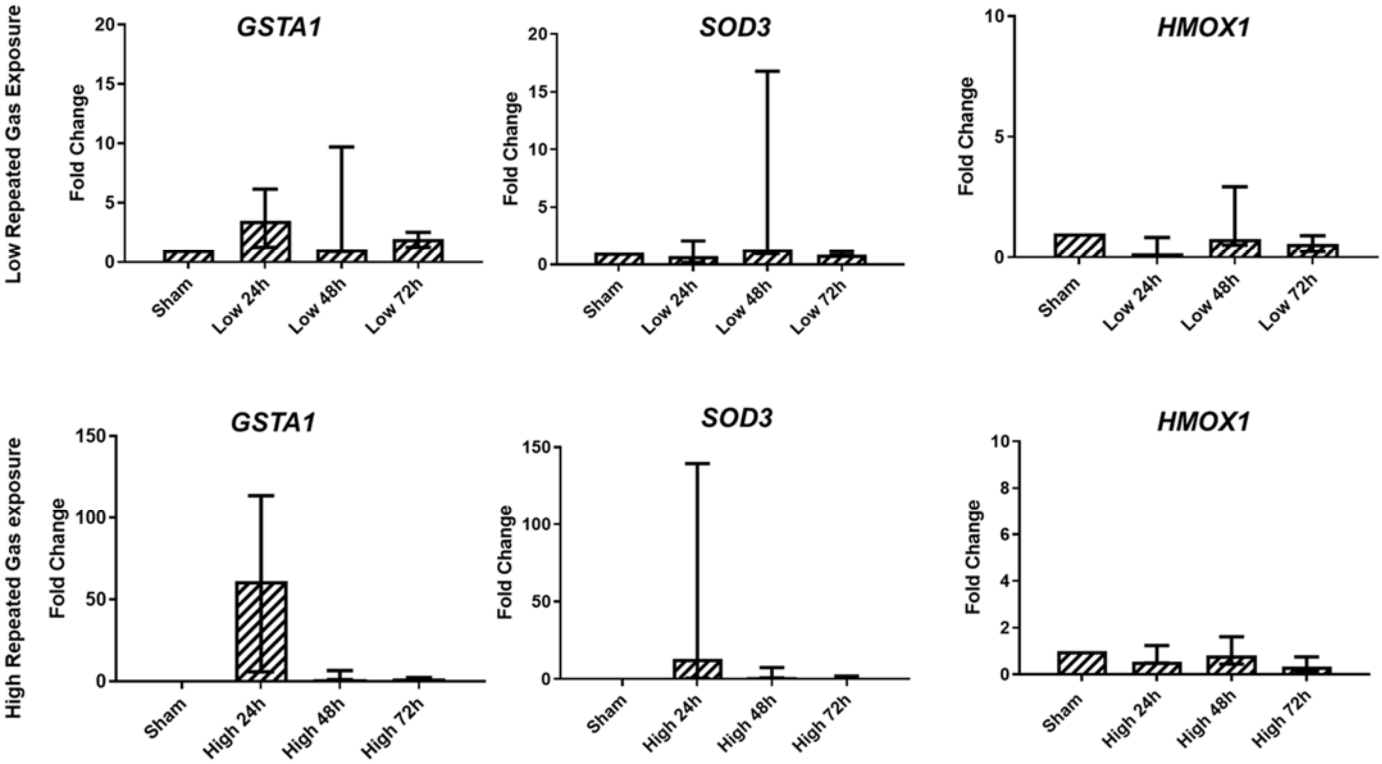


**Figure S2. mRNA expression of oxidative stress markers following repeated exposure to low and high doses of $NO_2$ and $SO_2$.** Fold change *of GSTA1, SOD3, and HMOX1* in bronchial mucosa models developed at air-liquid interface (bro-

ALI) following incubation for 24 hours (h) after 30 exposures to sham (control: clean air exposure); and gases: low ($NO_2$: 0.1 ppm; $SO_2$: 0.2 ppm) and high ($NO_2$: 0.2 ppm; $SO_2$: 0.4 ppm) doses for 3 time points (24, 48 and 72 h). Data presented as median and 25th -75th percentiles, fold change =2-ΔCt of models / 2-ΔCt of sham exposed bro-ALI. N=4 donors and n=2 replicates/Donor.

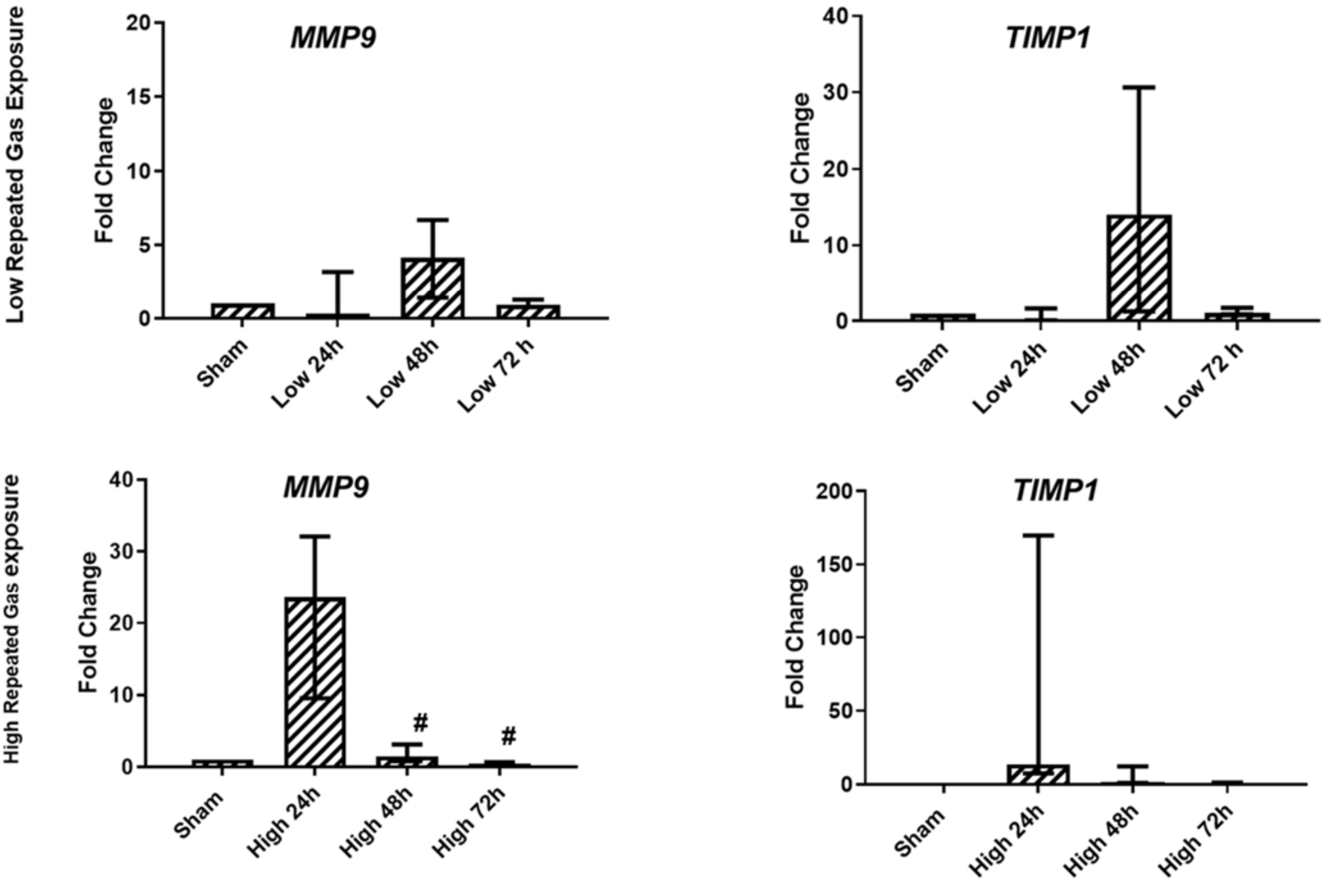


**Figure S3. mRNA expression of tissue injury/repair following repeated exposure to low and high doses of $NO_2$ and $SO_2$.** Fold change *of MMP9 and TIMP1* in bronchial mucosa models developed at air-liquid interface (bro-ALI) following incubation for 24 hours (h) after 30 exposures to sham (control: clean air exposure); and gases: low ($NO_2$: 0.1 ppm; $SO_2$: 0.2 ppm) and high (NO2: 0.2 ppm; SO2: 0.4 ppm) doses for 3 time points (24, 48 and 72 h). Data presented as median and 25th -75th percentiles, fold change =2-ΔCt of models / 2-ΔCt of sham exposed bro-ALI. N=4 donors and n=2 replicates/Donor.

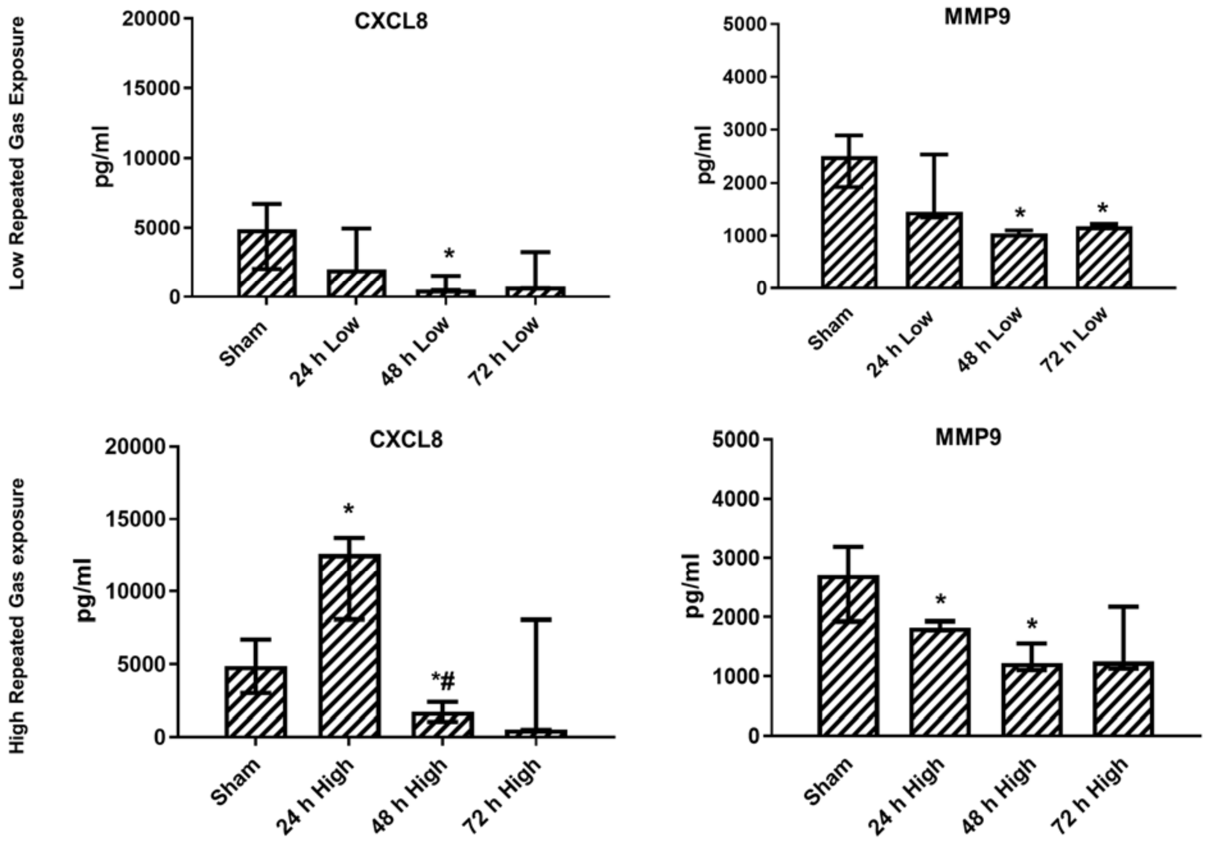


**Figure S4. Release of IL8 and MMP9 in basal medium following repeated exposure to low and high doses of $NO_2$ and $SO_2$.** Concentration of IL8 and MMP9 in TIMP1 in bronchial mucosa models developed at air-liquid interface (bro-ALI) following incubation for 24 hours (h) after 30 minutes exposure to sham (control: clean air exposure); and gases: low ($NO_2$: 0.1 ppm; $SO_2$: 0.2 ppm) and high ($NO_2$: 0.2 ppm; $SO_2$: 0.4 ppm) . Data presented as median and 25th-75th percentiles (N=4 donors and n=2 replicates/donor). $p < 0.05$ *: significant compared to sham, #: significant compared to exposed.
